# Supplementary figures and images for: Experimental investigation of orangutans’ lithic percussive and sharp stone tool behaviours
Source: PLoS One. 2022 Feb 16;17(2):e0263343. doi: 10.1371/journal.pone.0263343 (PMC8849460; doi:10.1371/journal.pone.0263343)

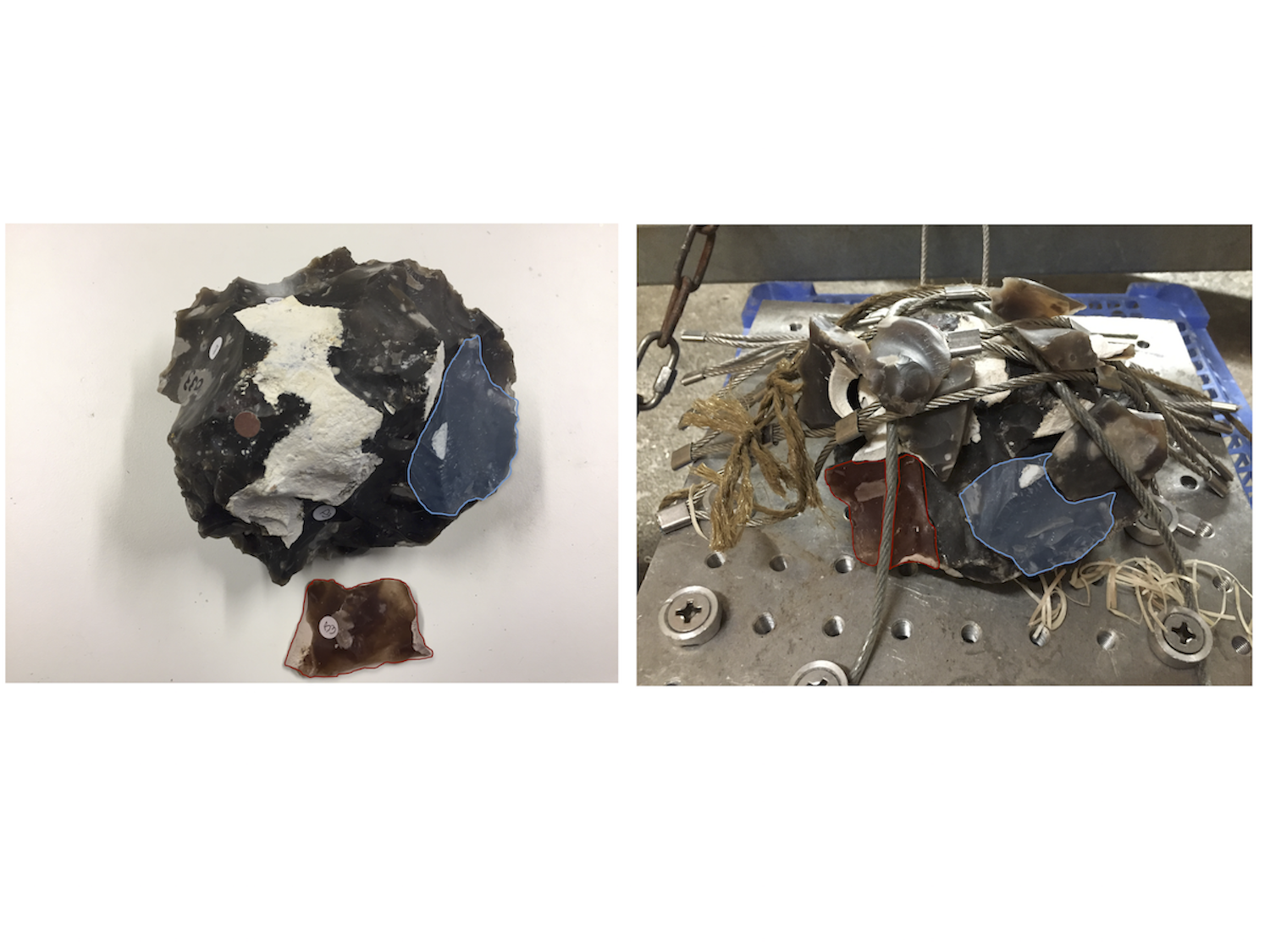

Supplement: S1 Fig — The left panel illustrates the core provided during the Flake Trading condition together with the two refitted flakes shadowed in blue and red. The right panel illustrates the fixed core as presented to the apes, with the two refitted flakes and the loose flakes placed on top of the core. (TIF) [file pone.0263343.s001.tif]

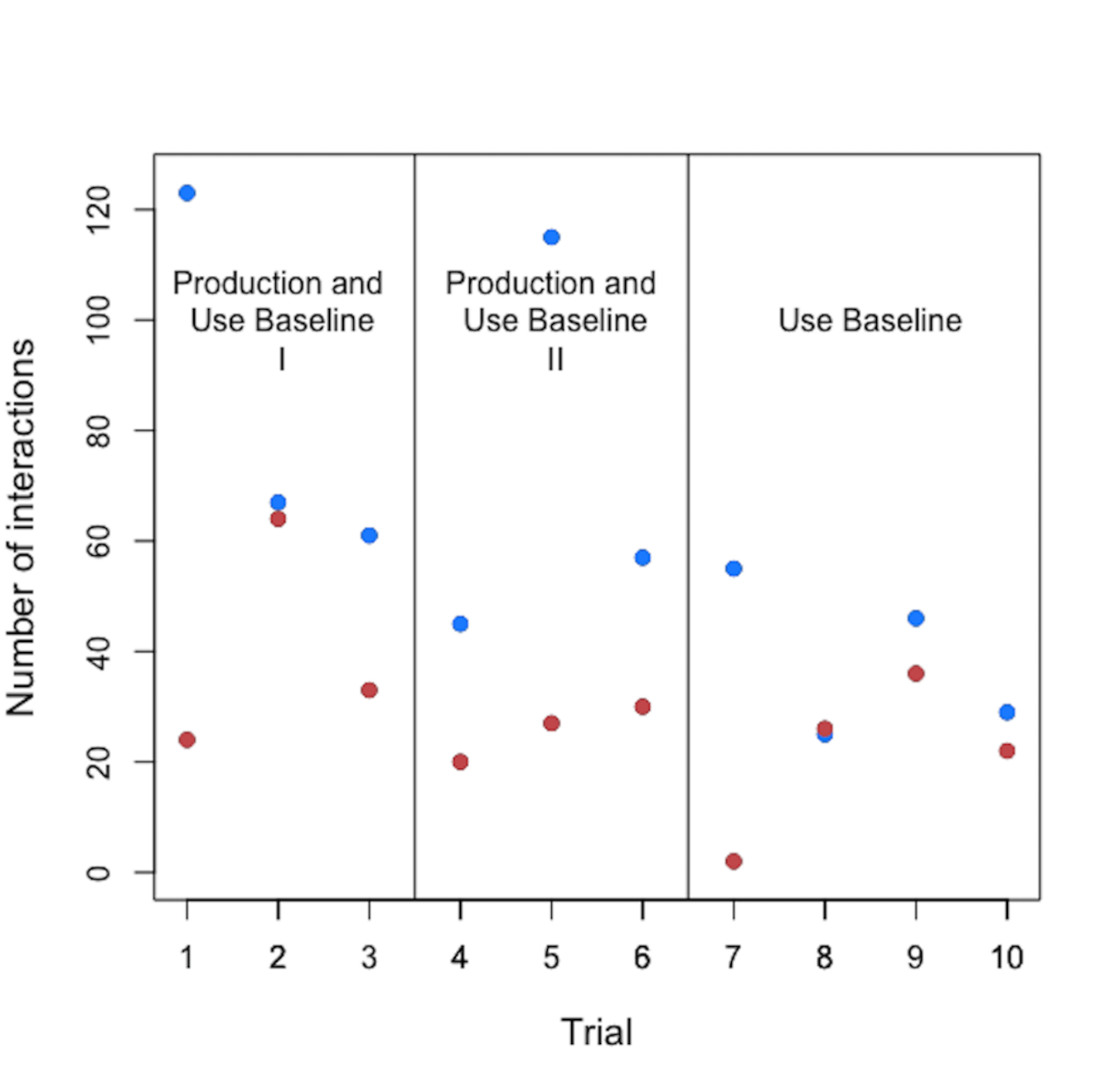

Supplement: S2 Fig — Number of interactions performed by each individual in each condition of Experiment 1. Blue dots represent the juvenile orangutan Loui and red dots represent the adult orangutan Matthieu. As there was no significant correlation between trial length and the number of interactions performed by the orangutans (Person correlation R = 0.016, p = 0.95), the results are displayed as sums of interactions rather than sums of interactions divided by trial length. (TIF) [file pone.0263343.s002.tif]

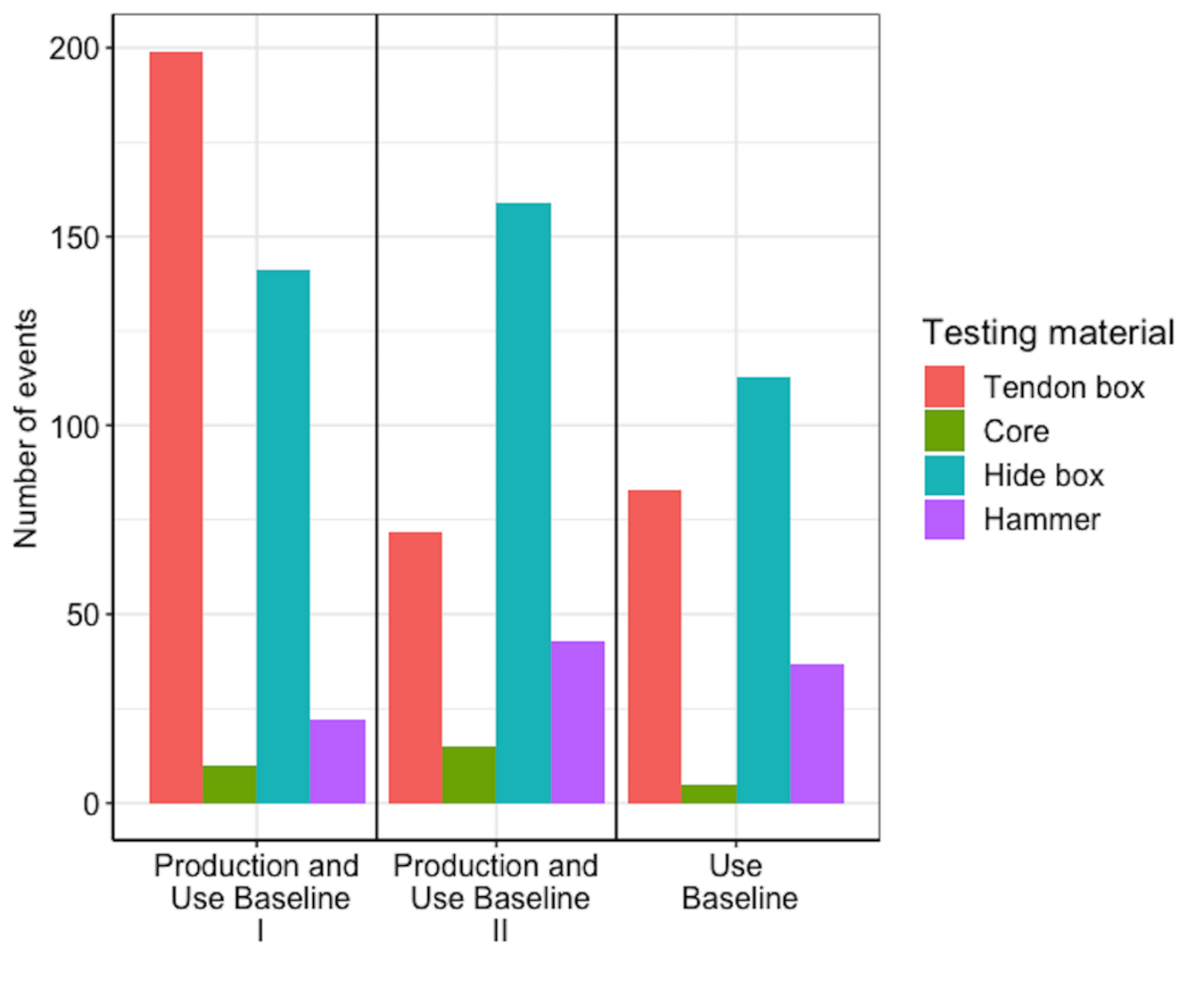

Supplement: S3 Fig — Number of interactions of orangutans towards the different testing materials in each experimental condition. Four of the interactions in the Use Baseline involved the human-made flake. In three occasions the flake was used to contact the drum and in one occasion to contact the core. The juvenile orangutan performed all the interactions involving the flake. (TIF) [file pone.0263343.s003.tif]

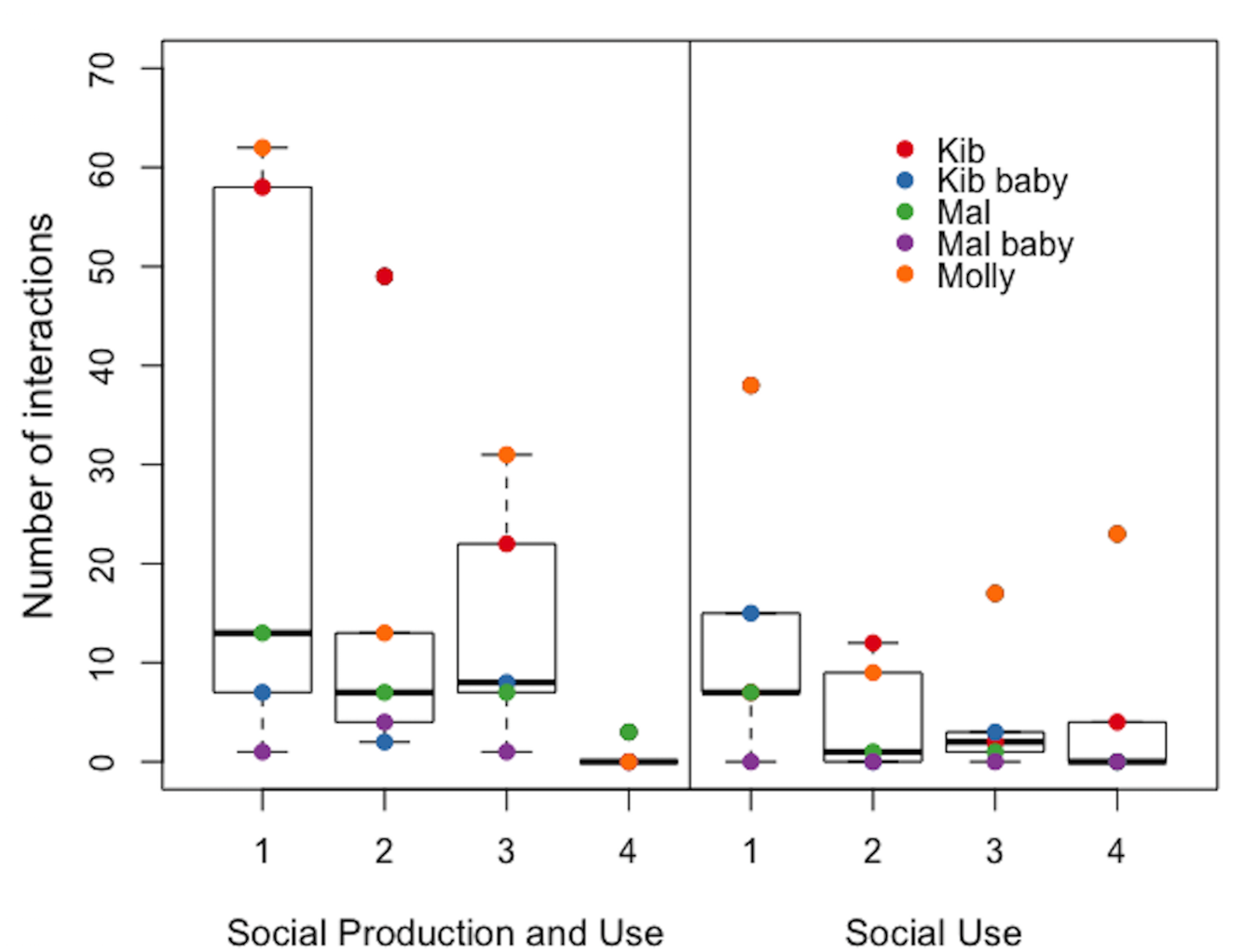

Supplement: S4 Fig — Each color represents a different individual. Bold horizontal black lines represent median number of interactions across individuals. Boxes represent interquartile ranges (IQR). (TIF) [file pone.0263343.s004.tif]

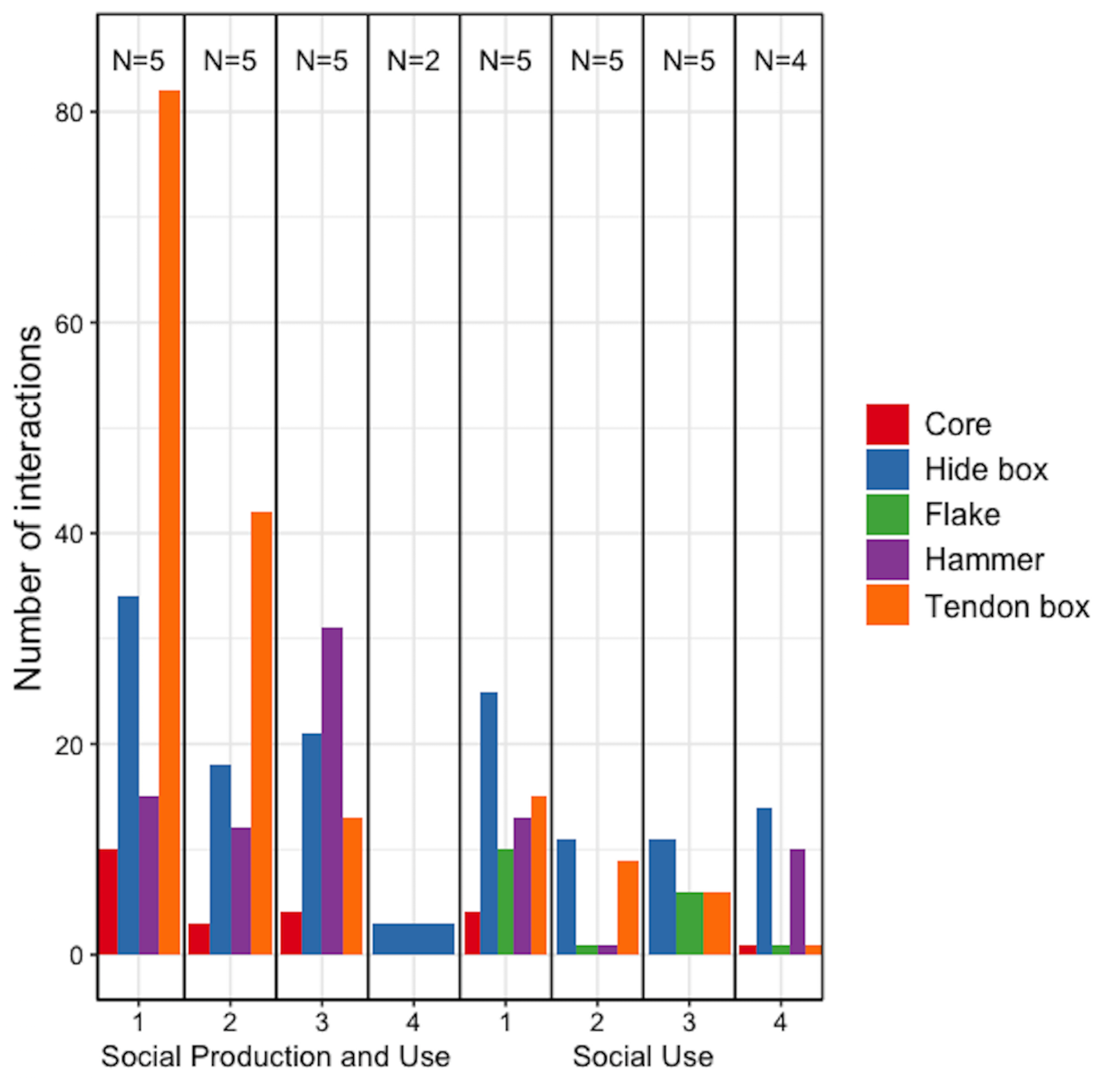

Supplement: S5 Fig — N represents the number of individuals tested in each trial. (TIF) [file pone.0263343.s005.tif]

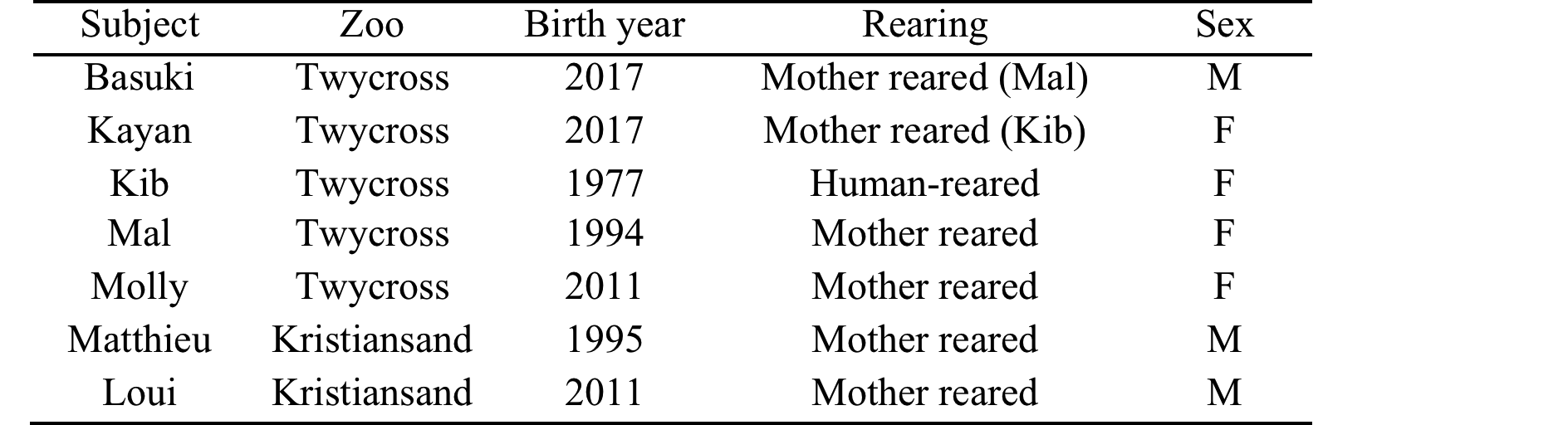

Supplement: S1 Table — (DOCX) [file pone.0263343.s006.docx]
