# Supplementary material for: Experimental investigation of orangutans’ lithic percussive and sharp stone tool behaviours
Source: PLoS One. 2022 Feb 16;17(2):e0263343. doi: 10.1371/journal.pone.0263343 (PMC8849460; doi:10.1371/journal.pone.0263343)
Supplement: S2 Table — (DOCX) [file pone.0263343.s007.docx]

| Testing material | Interact via | N | Total time  (seconds) |
| --- | --- | --- | --- |
| Touch tendon box | body part | 348 | 6573 |
| Touch tendon box | tool | 6 | 61 |
| Touch core | body part | 32 | 148 |
| Touch core | tool | 1 | 3 |
| Touch hide box | body part | 406 | 4638 |
| Touch hide box | tool | 7 | 54 |
| Touch hammer | body part | 107 | 1075 |
