# Supplementary material for: Experimental investigation of orangutans’ lithic percussive and sharp stone tool behaviours
Source: PLoS One. 2022 Feb 16;17(2):e0263343. doi: 10.1371/journal.pone.0263343 (PMC8849460; doi:10.1371/journal.pone.0263343)
Supplement: S3 Table — In parenthesis is the proportion of flakes exchanged from those provided. "+2" indicates that the refitted flakes were also exchanged. (DOCX) [file pone.0263343.s008.docx]

| Individual | Familiarization | Trial 1 | Trial 2 | Trial 3 | Trial 4 | Total flakes exchanged |
| --- | --- | --- | --- | --- | --- | --- |
| Loui | 10 (100%) | 6(100%) | 6(100%) | 6+2 (100%) | 6+2 (100%) | 38 |
| Matthieu | 10 (100%) | 6(100%) | 6+2 (100%) | 6+2 (100%) | 6+2 (100%) | 40 |
