# Supplementary material for: Experimental investigation of orangutans’ lithic percussive and sharp stone tool behaviours
Source: PLoS One. 2022 Feb 16;17(2):e0263343. doi: 10.1371/journal.pone.0263343 (PMC8849460; doi:10.1371/journal.pone.0263343)
Supplement: S4 Table — (DOCX) [file pone.0263343.s009.docx]

| Trial number | Duration (sec) | Behaviour | Number of strikes |
| --- | --- | --- | --- |
| 2 | 10 | Hit floor with core | 11 |
| 2 | 4 | Hit floor with core | 6 |
| 2 | 16 | Hit floor with core | 18 |
| 2 | 5 | Hit floor with core | 8 |
| 2 | 4 | Hit floor with core | 4 |
| 4 | 9 | Hit floor with core | 8 |
| 4 | 3 | Hit wall with core | 6 |
| 4 | 4 | Hit floor with core | 1 |
| 4 | 3 | Hit wall with core | 4 |
